# Active Learning Strategies in Veterinary Medicine: A Literature Review

**DOI:** 10.1155/vmi/7397383

**Published:** 2026-08-04

**Authors:** Bridget C. Garner, Paola Cazzini, Ricardo Marcos

**Affiliations:** ^1^ College of Veterinary Medicine, University of Georgia, 501 DW Brooks Drive, Athens 30602, Georgia, USA, uga.edu; ^2^ Royal (Dick) School of Veterinary Studies, University of Edinburgh, Easter Bush Campus, Roslin, Midlothian EH25 9RG, UK, ed.ac.uk; ^3^ School of Medicine and Biomedical Sciences, ICBAS, Rua de Jorge Viterbo Ferreira 228, 4050-313, Porto, Portugal

## Abstract

Many different active learning strategies that help students engage and interact with their own learning exist, and these range from simple, low‐technology, individual‐focused solutions to more complex, time‐intensive group activities that require extensive preparation and access to technology. While active learning is becoming increasingly emphasized in the veterinary curriculum, a robust and recent review describing the techniques used in the veterinary field is not readily available. PubMed and ERIC databases were searched using the terms “active learning/teaching” and “student centered/focused.” Ultimately, 147 references underwent full‐text reviews, with active learning techniques categorized into three primary domains: collaborative activities, individual tasks, and educational gaming and simulation. Features of techniques within each category are summarized, as are some of the advantages versus limitations, previously published student and instructor experiences, examples, and outcomes. While there are numerous published studies from the health professions, those focused on veterinary medicine are few and often limited to qualitative or descriptive data with survey‐based outcomes. While no method is a panacea for successful learning, our purpose was to highlight the existing best practices, delineating the status of active learning in veterinary medicine. More quantitative evidence on its effectiveness and impact in veterinary medicine can be gathered in the future to further guide teachers and educators.

## 1. Introduction

Preclinical veterinary teaching, as well as instruction in other health professions, has historically relied almost exclusively on didactic lecture methods. In these teacher‐centered or passive learning environments, students have little opportunity to contemplate, apply, or reflect on new information like being passengers in a car driven by someone else. Some students, particularly surface learners who primarily focus on memorization, prefer this traditional approach. Facilitator questioning and probing methods are necessary to elicit responses and engagement during lectures [1]. In contrast to passive didactic lectures where the instructor primarily focuses on content delivery, active learning (AL) is a broad term that refers to any educational activity where students engage and interact with the learning process. In the car analogy, students participating in AL help to power and steer the vehicle. The instructor and students work cooperatively to facilitate learning through three interrelated components: intentional engagement, purposeful observation, and critical reflection [2]. Graffam describes that for learning to be active, learners not only need to do something, but they also need to reflect on what they are doing [2]. Four principles that lead to more effective learning include AL along with providing feedback, individualizing learning, and creating relevance [3].

Scientific evidence supports the shift toward AL. These methods have been shown to enhance knowledge acquisition [4, 5], improve long‐term retention [6], boost performance in the sciences [7, 8], and increase lecture attendance [5]. Additionally, there is a moderate correlation between the use of AL and higher scores on standardized exams [6]. Technological developments have further catalyzed this shift, providing a wealth of options and resources for AL.

The effectiveness of AL strategies varies across student groups. Learning through case‐based activities, games, and teamwork can selectively benefit students with lower academic achievement, improving their test scores [9, 10], and knowledge retention [11]. Notably, some AL activities appear to have the greatest impact on less motivated students [12], underscoring its potential to engage and support a diverse range of learners. Learning through cases, games, and the self‐directed approach is particularly effective in supporting students who already have some foundational knowledge, are more familiar with diverse teaching styles, and are therefore best used in later rather than earlier years [13, 14]. Finally, AL irrespective of the method used has been shown to be disproportionately beneficial for individuals from low‐income backgrounds and for underrepresented minorities [15].

Creating an environment that promotes critical thinking and deeper learning is fundamental to making a class “unmissable” [16]. A primary advantage of AL is its ability to stimulate student interest and sustain cognitive engagement. Physiological data support this; for instance, heart rate (HR) has emerged as a proxy for mental effort, with elevated HR correlating to higher levels of focus. In a study incorporating AL techniques, such as collaborative problem‐solving, data analysis, and think‐pair‐share (TPS) activities, researchers observed that while student HR naturally plateaus or declines during passive lecturing, it spikes during active segments [17]. Although these activities do not necessarily “reset” a student’s attention span for the remainder of the lecture, the consistent HR increases in both morning and afternoon sessions suggest that AL acts as a vital re‐engagement mechanism. While higher physiological arousal is not a direct measurement of knowledge acquisition, it indicates the attentional state necessary for meaningful learning to occur.

In 2015, AL was listed as one of the 9 pedagogical principles for building a modern veterinary curriculum [18]. In that same year, the Competency‐Based Veterinary Education (CBVE) Working Group was established. The CBVE framework that was developed is an outcome‐focused, learner‐centered approach that represents a major pedagogical shift in veterinary education and outlines nine measurable Domains of Competence that graduating veterinarians must achieve to be practice‐ready [19]. AL aligns with this modern CBVE, which emphasizes the domains of self‐directed learning (SDL), collaboration, and scholarship, as well as clinical reasoning and decision‐making.

Although there is broad consensus that AL is beneficial, several factors may oppose this change. Institutions theoretically favor such a transition, but financial constraints [20], along with reduced staff, larger class sizes, and a streamlined curriculum, especially in basic science, often hinder this shift. Teachers cite many obstacles, such as insufficient time, lack of pedagogical training, concerns about content coverage, and negative student evaluations [5, 21, 22]. In fact, teachers who try AL methods often revert to passive lecturing, thus continuing to teach as they were taught in their own education [23]. Students may also push back, as some dislike being forced to interact during lectures or complain about the lack of flow in active classrooms [5]. A hierarchical culture may also inhibit student participation [24], and access to digital tools or to technology resources may be uneven among students [25].

Papers focusing on the use of AL techniques in veterinary education have been published in recent years [26, 27], but most describe a single intervention and have qualitative outcomes. AL encompasses diverse methods including case‐based learning (CBL) and experiential learning, peer collaboration in problem‐solving, and project‐based activities, among others. Technology has further enhanced AL through tools such as audience response systems (ARS), vodcasts, virtual patient simulations, and online educational games. Since AL is a pedagogical principle considered foundational in modern, international, veterinary curricular development [18], and no single method should be viewed as a panacea for successful teaching in every population of students, veterinary medical educators may benefit from a comprehensive resource summarizing numerous strategies from a review of research evidence. Thus, the primary aim of this review was to create a list of AL strategies pertinent to veterinary medical education. Because universally established categorizations are lacking, a broad search approach was necessary to first identify and map these categories. By prioritizing the breadth of the search, we ensured that all major pedagogical categories were represented. We incorporated strategies that vary in cost, complexity, time utilization, and technological requirements, making them suitable for diverse veterinary curricula. While the Scholarship of Teaching and Learning (SoTL) literature provides a robust framework for AL, veterinary medical education is still in the process of building a specialized evidence base [18]. Consequently, this review draws on transdisciplinary SoTL research (such as medicine, pharmacy, nursing, and dentistry) to address the current gap between the widespread practical adoption of AL in veterinary medicine and the validation of these methods in the classroom.

## 2. Literature Review Methods

A search of the literature was performed utilizing “rapid review” methods, which assess what is already known about a topic while using simplified systematic review methods to search and critically appraise the research as determined by time constraints [28, 29]. After meetings among all authors, the relevant search terms were clarified, key databases were identified, and inclusion and exclusion criteria were established (Supporting 1). The initial search was conducted by all authors in the PubMed and ERIC databases between October 24 and 27, 2023, using the following broad Boolean terms: “active learning” or “active teaching” or “student centered” or “student focused” AND (veterinar∗ or medic∗). This approach allowed us to capture the primary landscape of AL without becoming focused on a single method. Aligning with previously reported methods [29, 30], only resources published in the English language and time‐limited to 2003 to 2023 were included in order to identify the most up‐to‐date literature. The ERIC database search was limited to peer‐reviewed journal articles only. This search strategy yielded 5242 articles.

**FIGURE 1:**
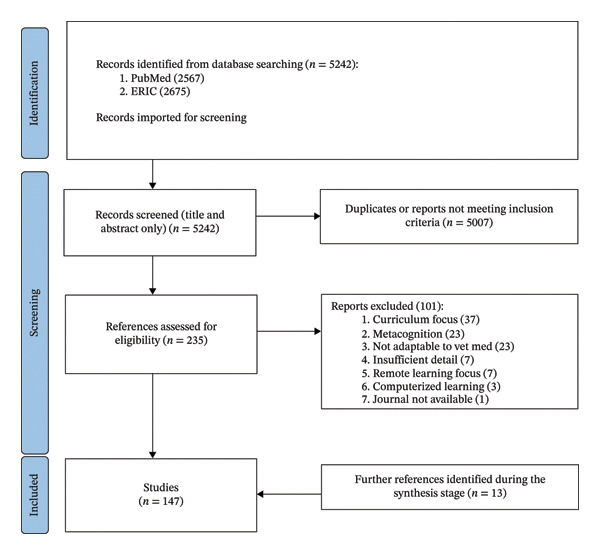
PRISMA flow chart depicting the search method used.

Citations were downloaded into a spreadsheet for sorting. Screen criteria were established and calibrated among the team through a series of pilot tests. After consistent mutual agreement was observed, the authors manually screened the literature search results independently, and discrepancies were resolved through discussion. Titles and abstracts of the retrieved records were screened to exclude duplicates and articles that did not meet inclusion criteria. There was no restriction on the study types to be analyzed; therefore, reviews, practice guidelines, and descriptive studies were included along with interventional research. Following the initial screening phase, 235 full‐text references were deemed eligible. These were examined more closely against inclusion criteria by each independent team member, with a subset verified by a second team member (BG) [31]. Because terminology can be heterogeneous, inclusion was not determined by keywords alone. Articles had to describe instructional method(s) that engage students in the learning process by involving them in meaningful learning activities and encouraging reflection on those activities [2]. A technique qualified for inclusion if it shifted the cognitive burden from the instructor to the student through interactive, collaborative, or inquiry‐based methods. This allowed for the inclusion of diverse methodologies provided that they moved beyond the traditional passive lecture format. During this level of review, an additional 101 references were removed from the study for at least 1 of 7 reasons (listed in the PRISMA flowchart, Figure 1). Papers were most commonly removed because they focused on the institutional or programmatic curriculum rather than on the individual (*n* = 37). Others either focused heavily on student metacognition (*n* = 23) or the techniques used were not readily adaptable to veterinary medicine (*n* = 23). Papers in which there was insufficient detail provided to recreate the activity (*n* = 7) or those in which the primary focus of the study investigated remote vs. in‐person learning (*n* = 7) were also removed. Finally, studies focusing on computerized learning that were not culled based on title and abstract (*n* = 3) or in which the journal was not available to any authors (*n* = 1) were excluded.

All 134 references that met the criteria for data extraction were added to an online reference manager system (Mendeley, Elsevier, London, UK). Each reference, including review papers, was then tagged by an independent reviewer with one or more primary tags mutually agreed upon by the group and further categorized as either a collaborative activity (where group activities are prioritized), an individual task (in which much of the activity is reliant on the individual rather than a group), or educational gaming and simulation. Papers could have multiple primary tags and thus be listed in multiple categories. These subjects were independently reviewed by each of the authors. Since methodological rigor regarding quality appraisal (QA) varies significantly across rapid systematic reviews, and appraisal of qualitative research using checklists meant for quantitative studies is also not straightforward, each article was evaluated for inclusion by each one of the authors instead of using a structured QA tool [28, 29, 32, 33]. This allowed for a nuanced evaluation of the pedagogical relevance and clinical applicability within a veterinary education context. To ensure a comprehensive review, the authors supplemented the systematic search with manual retrieval methods. This included snowballing (backward citation tracking) of all included studies and hand‐searching of expert‐identified literature. An additional 13 relevant articles were identified through manual searching and citation tracking. These were added to ensure comprehensiveness and contextual depth but were not part of the initial systematic screening process.

## 3. Active Learning (AL) Strategies

Our review identified several broad types of AL strategies we categorized as either collaborative activities, individual tasks, or educational gaming and simulation (Table 1). Across all categories, there was a limited corpus of veterinary‐specific studies, so sample group and size, study methods, and summarized outcomes from these veterinary studies can be found in Tables 2, 3, and 4. Most outcomes from these are qualitative, survey‐based results; therefore, studies from other health sciences that quantitatively capture increased student learning from each category are also listed. Finally, Table 5 briefly describes ways in which each strategy aligns with the core characteristics of AL. While these examples are not exhaustive, they highlight ways in which students engage with content, observe feedback, and reflect on their personal learning. A glossary of relevant terms can be found in Supporting Information 2. We summarize below the main features of each strategy and share outcome data, where available. At the conclusion of each segment, a specific use case example applying that strategy to veterinary medical education is listed. Guidance on the specific classroom context where these strategies could be effective is also provided.

**TABLE 1:** Distribution of active learning categories and primary strategies.

Category	Subcategory	Study count (*n*)	Top strategies identified
Collaborative	Peer‐to‐peer	18	Jigsaw groups, small group cases, group seminar preparation, student tutors, writing practice tests, peer grading
	Case‐based	23	Guided inquiry case discussions (in‐person and digital/online), virtual patients, blind cases, case cards
	Team‐based	13	Individual and team readiness tests, application scenarios
	In‐class activities	13	Think–pair–share, debate preparation, two‐envelope method

Individual	Interactive lecture	21	Polling, in‐class questions, muddiest point
	Flipped classroom	19	Interactive pre‐class work, pre‐class quizzing, microlectures
	Self‐directed learning	6	Predefined learning objectives, self‐study modules, structured logbooks

Educational gaming and simulation	Game‐based	22	Board games, card games, digital or online games, digital escape rooms, crossword puzzles, Jeopardy‐style games
	Simulation	20	Low‐ and high‐fidelity models, augmented reality, virtual reality,

*Note:* Total exceeds the number of included studies as studies utilizing multimodal strategies span multiple categories.

**TABLE 2:** Summary of literature supporting collaborative active learning strategies in veterinary and health sciences education.

Subcategory	Veterinary medicine–specific evidence	Veterinary medicine sample group	Veterinary medicine methods	Primary Outcome(s)	Supporting SoTL literature in health sciences education for student learning
Peer‐to‐peer	McMichael et al. (2021)	First‐year veterinary students, *n* = 152	Survey of student experience	Peer review improved work; multimedia improved communication and understanding	Gupta et al. (2021); Romeike et al. (2019); Sinnayah et al. (2019); Williams et al. (2018)

Case‐based	Marahrens et al. (2023)	Third‐year veterinary students, *n* = 247	Survey of student experience and assessment of completion attempts	High acceptance of digital format; improved task speed and accuracy across attempts; positive qualitative comments	Ali et al. (2018); Koles et al. (2005)
	Cardamone et al. (2022)	First‐year veterinary students, *n* = 98	Survey of student confidence	Positive shifts in postsurvey student motivation and confidence levels	Above
	Berrian et al. (2021)	Second‐year veterinary students, *n* = 110	Survey of student opinion and perceived competence	Improved scores in post‐questionnaires about perceived competency; high engagement	Above
	Crowther et al. (2016)	First‐year veterinary students, *n* = 121	Survey of student opinion	Students reported that CBL helped with material integration	Above
	Patterson et al. (2007)	First‐year and second‐year veterinary students, *n* = not provided	Course evaluations	Qualitative comments were positive.	Above

Team‐based	Hazel et al. (2013)	First‐year veterinary students and undergraduate students, *n* = 264	Survey of student experience and exam scores	Team scores outperformed individual scores; positive themes of knowledge retention and socialization; initial concerns about session length	Carrasco et al. (2018); Jost et al. (2017); Koles et al. (2005)

In‐class activities	N/A	N/A	N/A	N/A	Koklanaris et al. (2008)

*Note:* N/A: Not available from the literature search using the parameters described.

**TABLE 3:** Summary of literature supporting individual task active learning strategies in veterinary and health sciences education.

Subcategory	Veterinary medicine–specific evidence	Veterinary medicine sample group	Veterinary medicine methods	Primary outcome(s)	Supporting SoTL literature in health sciences education for student learning
Interactive lecture	Gordon et al. (2022)	Fourth‐year veterinary students, *n* = 84	Survey of student confidence	Activity enhanced clinical reasoning, problem‐solving, and communication	Gupta et al. (2021); Katsioudi et al. (2021)
	Berrian et al. (2021)	Second‐year veterinary students, *n* = 110	Survey of student perception	Improved scores on the postquestionnaire about perceived competency; high engagement	Above

Flipped classroom	Dooley et al. (2018)	First‐year veterinary students, *n* = 132, and undergraduate students, *n* = 282	Student grades, surveys, and focus groups	Higher grades and satisfaction scores for the flipped cohort.	Zhong et al. (2022); Hew et al. (2018)
	Moffett et al. (2014)	First‐year veterinary students, *n* = 197	Examination performance and survey of student perceptions	Traditional cohort achieved higher exam scores; flipped classroom was rated more positively	Above

Self‐directed learning	Gledhill et al. (2017)	Veterinary students, *n* = 1070	Survey of student experience	Common use of open educational resources; geographical inequalities persist	Yousaf et al. (2023); Murad et al. (2010)
	Dale et al. (2013)	Fifth‐year veterinary students, *n* = 75	Survey of student satisfaction, interviews, and focus groups	Logbook enhanced student confidence and competence; reliant on supervisor engagement	Above

**TABLE 4:** Summary of literature supporting educational gaming and simulation active learning strategies in veterinary and health sciences education.

Subcategory	Veterinary medicine–specific evidence	Veterinary medicine sample group	Veterinary medicine methods	Primary outcome(s)	Supporting SoTL literature in health sciences education for student learning
Game‐based learning	Loewen et al. (2023)	Third‐year veterinary students, *n* = 14	Survey of student experience	Effective for teamwork and a strongly favored activity	Dabbous et al. (2023); Gunter et al. (2022); Kanthan et al. (2011)
	Vitale et al. (2022)	Fifth‐year veterinary students, *n* = 49	Survey of student experience^∗^	Game format was more enjoyable; there was no difference in test scores.	Above

Simulation	Anderson et al. (2023)	Undergraduate students, *n* = 26	Short‐ and long‐term task performance scores	Simulation training contributed to similar performance scores as those trained on live animals	Staropoli et al. (2018); Cook et al. (2013); Evans et al. (2010)
	Madden et al. (2023)	Fifth‐year veterinary students, *n* = 32	Survey of student opinion and assessment of student performance	High learner satisfaction with simulator with similar performance between groups.	Above
	Marcos et al. (2023)^a^	Fourth‐ and fifth‐year veterinary students, *n* = 219	Survey of student confidence and experience	Student confidence improved; repeated practice was important	Above
	Marcos et al. (2023)^b^	Third‐year veterinary students, *n* = 231	Survey of student experience^∗^	No differences in final grades; students reported to learn more with the model.	Above
	Marcos et al. (2023)^c^	Second‐ and third‐year veterinary students, *n* = 129	Survey of student experience and confidence	Students rated the activity as essential; high examination pass rates	Above
	Stowe et al. (2022)	Third‐year veterinary students, *n* = 12	Verbal feedback	Highly positive comments led to expansion into the core course	Above
	Baillie et al. (2020)	Second‐year veterinary students, *n* = 32	OSCE performance	Model group performance was better than video	Above
	Read et al. (2016)	Third‐year veterinary students, *n* = 30	Survey of student confidence and OSCE performance	Model training improved performance	Above
	Modell et al. (2002)	Veterinary students, *n* = 90	Survey of student experience and examination performance	Simulation improved exam performance; student comments were positive	Above

Abbreviation: OSCE: objective structured clinical examination.

^a^Vaginal cytology simulation study.

^b^Clinical examination simulation model study.

^c^Cytology sample collection simulation study.

^∗^No statistically significant differences or increases in final examination or post‐test scores.

**TABLE 5:** Indicators of engagement, observation, and reflection within active learning strategies.

Strategy	Engagement	Observation	Reflection
Peer‐to‐peer	Explain concepts to a partner or small group	Listen to a peer’s logic	Evaluate one’s own understanding against a peer’s
Case‐based	Solve a real‐world medical scenario	Identify key variables and patterns in the data	Assess the consequences of the chosen solution
Team‐based	Prepare individually followed by collaborative problem‐solving and debate	Observe team dynamics and diverse perspectives	Debrief with the entire group or complete individual journal entries
In‐class activities	Participate in short tasks like think‐pair‐share	Notice immediate knowledge gaps during the task	Consider the discrepancy between initial independent thought and the collaborative conclusion
Interactive lecture	Respond to polls	See the real‐time response trends of the class	Reconcile perceived competence with actual performance
Flipped classroom	Review content before class and apply it in the classroom	Observe one’s own readiness level during class	Assess progress from before to after class
Self‐directed learning	Research and select resources and learning objectives (LOs)	Monitor personal learning pace	Evaluate learning progress through the accomplishment of LOs
Game‐based	Navigate mechanics and competition	Track scores and opponent moves	Analyze causality between specific decisions and game outcome
Simulation	Roleplay or interact digitally	Observe system responses to specific inputs	Compare the simulated experience to the theoretical

### 3.1. Collaborative Activities

#### 3.1.1. Peer‐to‐Peer

Peer‐to‐peer learning (PPL, also known as peer‐assisted learning) is the process of peers learning with and from each other. In these scenarios, students within the same program, but not necessarily at the exact same level of the program [34], use their own abilities and knowledge to help others learn. General perceptions of PPL indicate that students may be more apt to provide on‐level or relatable explanations of the material than experts, and students may be more willing to ask questions to peers in small groups when clarification is needed [35]. Although not unique to PPL, the act of teaching others can also deepen students’ understanding. To explain a concept effectively, the instructor must have a clear grasp of it. Moreover, the process of explaining can reveal gaps in the instructor’s own knowledge or clarity, which they may then seek to address.

PPL activities can be adapted to small or large classrooms and can range from simple discussion to high‐tech formats using medical simulators. Students can be allowed to select their own groups [36] or be assigned by the instructor [35]. Groups may be encouraged to allocate roles to each member (e.g., leader, presenter, timekeeper, cheerleader, recorder, researcher) to accommodate a wide range of abilities and personality types, as well as to keep each other accountable [35, 37]. While groups with more than 5 members have been positively described [35, 38, 39], most reports suggest that limiting group size to 4‐5 students is optimal for student engagement [37–41].

**FIGURE 2:**
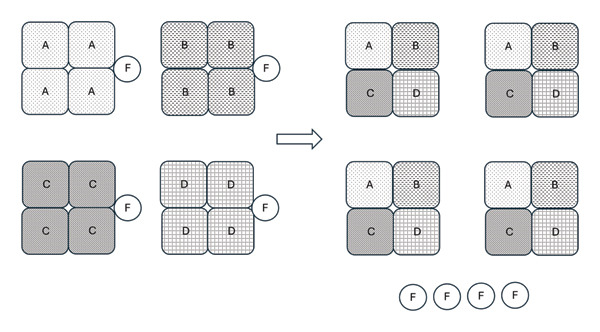
Illustration of the jigsaw or expert groups teaching method. Each student is assigned a different segment of a topic and after individual preparation joins an “expert” group to collaborate with peers studying the same content (left side). Facilitators (F) may assist with the discussion in these groups. After gaining a deeper understanding, students return to mixed groups (right side) to teach their segment to others, and facilitator participation is limited to clarifying points the student experts cannot explain.

PPL can remain within the designated small groups for the entire activity, or it can extend to others in the classroom in a variety of ways. Small groups can be assigned different subtopics on which to focus their attention and then present their content to the entire class [42, 43]. Similar to PPL is the jigsaw scheme (Figure 2); in this case, each student within a group is assigned a different task or topic, thereby dividing up parts of the subject or problem like puzzle pieces. For the duration of the activity, they are labeled as the “expert.” Time is initially set aside for individual study and focused preparation. Then, “experts” on a single topic from each group convene to master the concepts together. Following discussion that is typically facilitated by an instructor, who may be a faculty member or a postgraduate student [44], each “expert” returns to their home group, where they will serve as the peer instructor for the remainder of the session. Students reported a high level of student satisfaction with expert group activities [41]. These activities enhanced their understanding [45] and communication skills and helped with overcoming shyness and hesitation in class; however, participants also perceived it as time‐consuming, and members with a lack of interest could dampen the enthusiasm of others [39]. Some students reported lower satisfaction compared to faculty‐led sessions, possibly due to perceptions that peer explanations were less effective or because the method required more preparation time [46].

These activities are not limited to in‐person interactions. For example, students’ verbal explanations of topics can be recorded and then reviewed later by their peers [47]. Alternatively, students can write multiple‐choice questions (MCQs) that are then shared with their peers to assess learning [6, 48]. The latter scenario is anticipated to be of more benefit if students have participated in question‐writing training and have had the opportunity to practice writing questions that are subsequently edited for feedback.

PPL learning is an effective method of AL in medical education [34, 35, 49]. However, these new learning techniques may incite student anxiety [35], particularly if there is concern that the PPL format will not prepare them for the exam. As a result, the assessment content and format must align with this approach.

*Veterinary use example: In a Virology course, the class is divided into “expert” groups where each group focuses on one specific feline virus (e.g., FeLV, FIV, or FIP coronavirus). Students then move into mixed groups where they must teach their peers about their designated virus to collectively build a comprehensive diagnostic and vaccination plan for a cat shelter. While this approach necessitates more intensive initial instructional design and longer classroom blocks than the standard lecture format, it is highly recommended for multifaceted subjects (e.g., infectious disease) where the primary learning objective (LO) is the application of knowledge to clinical case management rather than rote memorization.*

#### 3.1.2. Case‐Based Learning (CBL)

Veterinary students routinely spend a portion of their training in the clinical setting. Case numbers and variety impact the type of cases a student will see. CBL can supplement these experiences and impart problem‐solving skills without the need for contact with real patients. CBL can highlight the clinical relevance of material covered in theoretical lectures, while also enhancing motivation and improving information recall by fostering deeper connections between concepts. CBL can be adapted to a variety of timelines and settings ranging from a portion of a lecture to the entire class period, small group exercises to whole classroom discussions, and activities requiring no technology to computer simulations. Conceptually, CBL differs from problem‐based learning. The former is more organized, and students have prior knowledge of the pathophysiology of the disease before being presented with the case, whereas in the latter, the activity starts with a problem that prompts students to search for information [50].

CBL is an effective way to engage and teach students in a variety of medical settings [13, 51–56]. Case modeling (where the instructor verbalizes the cognitive process involved in clinical case management) can provide a useful example of how to work through a case [2], and allowing students to design the case prompts, or triggers, can improve comprehension and critical thinking [57]. Veterinary students reported increased confidence and understanding of biochemical concepts [51] after utilizing clinical case studies, and others felt that case studies were relevant to real‐life scenarios, promoted AL, and assisted with integration of basic and clinical subjects [55].

CBL may not require access to technology. For example, case cards can be “low‐tech” [58]. A set of student cards is created such that only pertinent case information is included. Facilitators get separate instructor cards that contain student prompts, prioritized differential lists, management plans, and clinical pearls with important points that all students need to consider. If access to technology is not limited, then QR codes on the student cards can direct learners to open educational resources (OERs). Some of these resources are available in the veterinary field (e.g., https://www.veteriankey.com/clinical-cases/ or https://www.eclinpath.com). The use of those resources was boosted by the COVID‐19 pandemic, in which WeChat, eLearning, and Zoom online platforms were used for delivering CBL [59]. The specific use of digital cases has been described by Marahrens et al. [60], who reported increased student competency in discriminating blood cells of various species, with positive feedback.

There are limitations that instructors should consider when implementing CBL activities. Vitale et al. [53] compared the utility of case discussion to board games with veterinary students. Both methods received positive evaluations, but the board games allowed more interaction between students (rather than between the student and the instructor with case discussions) and encouraged the students to take responsibility for the cases in the game and make their own decisions. In other scenarios, students participating in simulations scored better on an examination than those who completed case discussions [61].

Different student populations may have variable opinions about this technique. Large group case discussions can prompt some students not to participate or share their thoughts [11]. Furthermore, group dynamics and peer dominance may impact students’ perceptions of CBL [13]. In one comparison, female students were more critical of the group dynamic when evaluating the utility of CBL [13]. This method may selectively benefit the lower academically achieving students. For instance, students whose mean exam performance in prior courses was below the median scored significantly higher if they participated in case‐based activities compared to those who did not [9]. Students in later stages of training enjoyed the case discussions more than first‐year students, because they had more foundational knowledge and were more used to this teaching style [13].

While limitations exist, combining CBL with other techniques is likely to outweigh the listed limitations. Instructors have reported that CBL helps convey important points that may otherwise be ignored, such as the utility of basic science concepts and their role in successful treatment and care of patients [62]. Veterinary pathologists have described using this technique and suggested that the ideal is to combine advanced preparation by the students (e.g., by flipped classroom [FC] learning) and delivery of fundamental concepts by the instructor prior to the case discussions [54].

*Veterinary use example: In a small animal Internal Medicine course, students are presented with the medical history and physical exam findings of a 9-year-old Beagle with polyuria and polydipsia (excessive urination and drinking). Working in groups of five, students must analyze the case to determine which diagnostic tests to order (e.g., serum biochemistry, urinalysis) and subsequently interpret the results to differentiate between diabetes mellitus and hyperadrenocorticism. The instructor facilitates by providing new clinical information as the students progress through their diagnostic plan. This approach is less resource-intensive than simulation but offers more realistic clinical preparation than traditional lectures. Late preclinical or early clinical year students likely have the foundational knowledge to benefit from this method most while they practice data interpretation and diagnostic test prioritization.*

#### 3.1.3. Team‐Based Learning (TBL)

TBL is a small group teaching method that uses the same student groups for a long period (often the entire semester) to accomplish a recurring sequence of activities [63–65]. This process relies on 3 phases that recur in sequence: (1) student individual study; (2) individual and team readiness testing to promote accountability; and (3) clinical application and discussion period. Within TBL, there are also chances for reflection, an integral aspect of learning. Some have encouraged providing additional opportunities for students to reflect on their own learning in TBL, such as in the form of repeated journal entries [66].

Students interact with their peers more than they interact with the instructor in this method [65], and one facilitator may oversee several groups in a large lecture hall [64]. As a result, facilitators are encouraged to have prior TBL training to help them anticipate and maneuver noisy and high‐energy environments [64] while also maintaining their own silence when students work through obstacles [67]. Quality facilitation, including knowing when to direct discussion, how to ask neutral and open‐ended questions, and how to reserve time for closure, is key to successful TBL [64]. As high‐functioning student teams are also a requirement, substantial student orientation to TBL may be necessary [64]. Furthermore, student groups may work best when specific traits have been considered during group development. For example, some recommend purposefully mixing students of different experience levels and educational background, as well as prioritizing gender and ethnic diversity when groups are formed [64].

TBL effectively helps students develop clinical decision‐making skills while working collaboratively [68–70], but one limitation is that its utility for teaching facts or knowledge concepts may not exceed that of traditional methods [11, 68]. Similar to other methods of group learning, TBL may selectively benefit lower academically achieving students, since those in the lowest quartile have shown the greatest improvement in retention of knowledge [11].

*Veterinary use example: In a semester-long Pharmacology course, students are assigned to permanent teams of five members. For one module (e.g., anesthetic drugs), students first complete individual preparation followed by an individual readiness test (iRAT). Next, the team immediately completes a team test (tRAT), where they must reach a consensus on drug mechanisms and side effects. The remainder of the session is spent on case application, such as designing an anesthetic protocol for a surgical patient with pre-existing renal disease. After the discussion, students complete an individual writing exercise reflecting on the activity. TBL is recommended for content-heavy courses where student-to-instructor ratios are high, as it allows a single facilitator to manage a large cohort. Educators may choose TBL when the goal is not only content mastery but also the development of professional soft skills, such as conflict resolution and group consensus building.*

#### 3.1.4. In‐Class Activities

Options for in‐class activities are numerous, and experienced instructors recommend performing a variety of these activities to maximize benefits [6]. Some, such as in‐class debate, require intense student preparation ahead of class, which leads to higher test scores compared to those who solely prepare for lecture [71]. Debate also encourages teamwork, inspires camaraderie, and provides a forum for controversial topics [71].

TPS is commonly used in‐class activity [72]. In TPS, students are initially given a scenario or question to consider independently. After a designated window of independent work or thought, students pair up with another student to discuss their responses. They may be asked to achieve a single, collaborative response, produce a list of multiple responses, or they may be asked to take on different roles (such as that of listener vs. talker) [72]. At the conclusion, pairs share their thoughts with the rest of the class. In medical education, this technique has been shown to work well with clinical case interpretation [72]. Unequal participation between paired students, personality conflicts, and time constraints can hinder successful TPS [72].

The two‐envelope method has been used in medical education with positive outcomes [73]. With this simple method, students are assigned reading and self‐study prior to class, and upon arrival, they are handed two envelopes. The first contains a quiz they complete independently and return to the envelope that is subsequently sealed. The second envelope containing the same test and additional clinical case information is then opened, and both are discussed within student groups, followed by a faculty‐led discussion. Benefits include increased time efficiency of faculty discussions as students have already completed self‐learning, group learning, and clinical integration steps. Students learn complex subjects on their own, and they also gain knowledge through interaction with peers [73]. In addition to these benefits, early testing improves learning [73] and has been shown to improve the quality and quantity of preparation [74].

*Veterinary use example: During a didactic Clinical Pathology lecture, the instructor displays a high-resolution image of a blood smear from a horse. Think: Students are given 60 s to individually identify the leukocyte present. Pair: They turn to their neighbor to compare observations and debate the cell identity. Share: The instructor asks a few pairs to explain their reasoning before revealing the correct answer. This strategy is ideal for high-enrollment foundational courses where students are hesitant to voice initial opinions to the large group. It can also be effective as a diagnostic tool for the instructor; by surveying the room after the “Pair” phase, the educator can immediately identify and correct collective misconceptions before moving on to more complex material.*

### 3.2. Individual Tasks

#### 3.2.1. Interactive Lecture

Lectures are widely used in the medical world as a form of teaching. Despite being typically passive learning, small modifications can improve student engagement and deeper learning. Student attention peaks at the beginning and end of the session. Thus, stating clear goals, providing a lecture roadmap, and asking about previous knowledge at the beginning of a lecture [75] are advised. Furthermore, ending a lecture on time and circling back to previously stated main points in the concluding remarks are also useful [75].

Collaborative activities (e.g., TPS, debate, two‐envelope method), as well as more individual tasks, can be done. While there are numerous examples, individual tasks include manipulating Play‐Doh to understand 3‐dimensional structures [76] and the “muddiest point” activity where students (often anonymously) tell the instructor what topic is currently confusing them most. Incorporating AL strategies into lectures inevitably takes time, which could otherwise be used for didactic teaching. However, the time allocated for such activities does not need to be great and can constitute an overall small portion of the total lecture time [22]. This time is never wasted, since the attention and engagement increase in those periods [17].

Regardless of the technique(s) used, alternating between short, instructor‐led mini‐lectures and individual, student‐driven in‐class activity phases provides many benefits, including maintaining attention span [77]. This is the main principle behind the “Sandwich model,” with the bread symbolizing the beginning and the end of the lecture, and the meat, cheese, and vegetables symbolizing the various passive and active components of the lecture [77].

One way to increase engagement in lectures is by using questions (Table 6). Indeed, when the audience knows they may be asked questions, it changes the way they listen [2]. Besides traditional dialog, questions can be answered using electronic systems or other methods like colored cards [3]. Questions can also be asked using polling or ARS, interactive technologies that allow large groups to answer questions, often anonymously, in real time. ARS can foster discussion, promote collaboration, and encourage broad student participation in a supportive environment. They help elicit diverse perspectives, enhance material retention, and provide real‐time assessment of student learning. In addition to real‐time polling, ARS can be used for formative assessments [78, 79]. Additionally, ARS can gauge student attitudes, improve class attendance, and bring an element of engagement and excitement, even in large lecture settings [78]. Several studies have reported positive student feedback about ARS and their ability to enhance engagement and enjoyment [79–81].

**TABLE 6:** Types of interactive lecture questions (adapted from Graffam 2007 and White 2011).

Type	Description	Pros	Cons
Open	Question posed to the whole group.	Encourages broad participation; allows for a range of answers and opinions.	Can feel rhetorical; may go unanswered if students are unsure or hesitant.
Targeted	A specific student is called upon to answer.	May help students feel seen and valued; gives quieter students a voice.	Can cause anxiety or discomfort, especially in shy or unprepared students.
Follow‐up targeted	A second student is asked to respond to or build on the first student’s answer.	Encourages peer dialog; may lead to deeper exploration or even debate.	Risk of putting students in an uncomfortable position; could lead to tension between peers.
Connection question	Links two or more topics or concepts.	Helps students make meaningful connections; promotes integrated understanding.	May be challenging for students if prior knowledge is patchy or undeveloped.
Pause and clarify	Lecturer pauses to allow students to discuss a concept with a neighbor.	Encourages active processing of difficult material; supports peer learning.	Requires time; may disrupt flow if not well‐managed.
Spectrum model	Students physically arrange themselves to stand for parts of a process or range.	Makes abstract concepts more concrete; encourages movement and collaboration.	Can be time‐consuming or chaotic; students may feel self‐conscious or competitive.

ARS can work with infrared (IR), radio frequency (RF), or wireless (Wi‐Fi) technology; the latter is now more widely used. Historically, remote controls (Clickers) [78] or personal digital assistants [82] were distributed to students at the beginning of classes. Advantages were that students did not have to purchase their own devices, yet they benefited from enhanced engagement. However, there were limitations, such as the upfront cost, time spent distributing and collecting, issues with malfunctioning, and the increased time required for instructors to set up questions [78, 81]. Even with these limitations, Clickers were used by teams in jeopardy‐style games that students found engaging, fun, and collaborative [83].

Now, smartphones or tablets are widely used for ARS. Software and applications, ranging in price from free to relatively expensive, can be used by the instructor to create different types of questions. Question formats can vary from MCQ, open‐ended, closed‐ended, true/false, and image‐based. Answers can be reviewed in real time, thus providing the instructor with data on the general understanding of the topic while giving immediate feedback to students. Questions can also be used to generate discussions and debates.

Multiple ARS platforms such as Socrative (Mastery Connect, Salt Lake City, USA), Wooclap (Wooclap SA, Brussel, Belgium), Poll Everywhere (Poll Everywhere Inc., San Francisco, USA), and VotAR (libre‐innovation.org, Compiègne, France) exist. These offer interactivity with minimal technological barriers [80, 81]. While some of these systems require students to use devices such as smartphones or computers to answer questions (e.g., Socrative, Wooclap, Poll Everywhere), others only require the use of devices by the instructor (e.g., VotAR, in which answers are cast by the audience by displaying unique, pre‐printed symbols, which are photographed and counted using augmented reality). When students’ perception of three ARS systems (VotAR, Socrative, and Wooclap) was compared, Socrative and Wooclap were favored; however, systems such as VotAR may be considered for large classrooms with a small budget or fewer technology resources [80].

*Veterinary use example: In an Oncology lecture about mast cell tumors (MCTs), the instructor presents a clinical scenario involving a canine patient with a 2-cm cutaneous mass and a cytologic diagnosis of MCT. Using Poll Everywhere, individual students are asked to select the most appropriate next step in clinical staging: a) immediate wide surgical excision, b) fine-needle aspiration (FNA) of the regional lymph node, or c) abdominal ultrasound and thoracic radiographs. The instructor displays the live poll results to see whether students prioritize local treatment (surgery) or systemic staging (searching for metastasis). If the majority of the class votes for surgery without staging, the instructor can immediately address the risk of clean surgical margins being irrelevant if systemic spread is already present. This strategy is effective for complex, decision-based topics where there is a common “trap” or incorrect clinical instinct. It can also be impactful for large group settings where individual feedback is otherwise impossible.*

#### 3.2.2. Flipped Classroom

In the traditional lecture model, new content is delivered during class, with students expected to consolidate their understanding through review after the session. In contrast, the FC presents foundational or lecture content before class, reserving in‐person sessions to increase comprehension through AL. Materials that students review prior to class are versatile and may include recorded lectures, textbook chapters, videos, clinical case reports, and wikis, among others. Recording lectures or preparing the material to be reviewed before class may be time‐consuming for the instructor initially, but time may be saved in future years by re‐using, or simply updating, these resources. One study comparing medical students receiving in‐person lecture to those receiving the same material in video format revealed similar test scores without significant differences [84]. This demonstrated that using videos in the FC approach does not compromise student learning, and instructor classroom time can be devoted to AL methods [84]. However, the quality of the instruction materials depends on the instructor [85]. One of the big advantages of independent, pre‐class content review is that students can learn at their own pace, gaining the ability to control the speed and bookmark sections of new material being presented, and are able to review concepts before and after the activity [69].

With the content covered before class, in‐person time can be used for a variety of AL activities such as discussion, CBL, group projects, problem‐solving, and reflection exercises [69, 86] promoting cognitive development [85]. While working through clinically relevant problems (e.g., CBL), students can use online didactic materials, books, or other resources for support [69], applying and consolidating knowledge. Novel teaching styles can be adopted, and students may have more student–tutor interaction during face‐to‐face sessions compared to traditional lectures [25].

Some studies suggest that FC improves performance and satisfaction, although its success depends on student motivation, independence, and proper scheduling [25, 87–89]. In undergraduate medical education, FC has been very well received in both preclinical and clinical courses [90]. However, some studies have shown mixed results, with FC leading to superior outcomes in some cases but not universally [87, 91]. FC correlated with better student performance in a cohort of biochemistry students [92], yet traditional methods were linked to higher test scores for veterinary students compared to those in the FC [91].

Downsides of FC are that students may arrive unprepared for class sessions [90, 93] or may not have access to reliable technology to review the material in advance [25]. Others might shy away from active social interaction and prefer to work in isolation and more efficiently, or might miss the traditional interaction with teachers and peers during the material review stage [89]. A short introduction with a microlecture going through the main points of the pre‐class material could mitigate student disengagement [86], and this could be followed by a brief question‐and‐answer session. The increased preparation time for instructors and potentially a need for multiple mediators during AL activities, the unsuitability of existing teaching spaces (with forward‐facing seats), and large class sizes may also be limitations [36, 93]. Overall, the benefits of FC in healthcare professions are considered to outweigh limitations [87].

One key consideration with the FC approach is the time available to students. To prevent overwhelming them, time for reviewing FC material must be incorporated into their timetable, just as time for AL activities is. If students are expected to study independently outside scheduled sessions, they may become overwhelmed, especially if multiple instructors in a team‐taught course simultaneously adopt the FC approach. As overall course time is limited, allocating time for both content review and AL activities may reduce the opportunity to cover all intended material.

“Blended learning” represents a midway approach between traditional lectures and the FC, combining face‐to‐face instruction with online content, and several variations have been described [94, 95]. It maintains student–teacher interaction and peer learning while offering flexibility by reducing in‐class hours. Shifting some content online can free classroom time for deeper engagement and interactive learning experiences [96]. A 2017 study replaced ∼ 50% of a pathology course with edited lecture excerpts, animated slides, and interactive quizzes [96]. The remaining in‐class time focused on overview lectures and technology‐enhanced formative assessments, offering progressive feedback and increased engagement. Online module usage declined throughout the course, but students who completed at least 90% scored significantly higher. Blended classroom approaches have been used in laboratory classes, incorporating digital slides [36, 94], also with good results. In a study comparing traditional lecture to FC blended learning, the latter was associated with higher final course scores, suggesting a blended approach combining virtual and in‐class teaching leads to higher knowledge retention [94]. While effective, the blended approach is not universally liked, mainly due to reduced student–teacher interaction and connectivity issues [96].

*Veterinary use example: In an Endocrinology course, students are assigned pre-class modules consisting of instructional videos about thyroid hormone signaling and the typical laboratory test results and clinical symptoms noted with thyroid disorders. An online quiz must also be completed prior to class. When students arrive for the in-person session, the instructor provides a brief (e.g., 10 min) recap. The remainder of the class period is dedicated to the review of physical exam findings and analysis of laboratory test results from a series of patients. This model works well for technical labs or clinical rotations where student-to-instructor ratios are low, but it can also be used in didactic courses where foundational knowledge is needed before discussion or application can occur. It is most effective when the pre-class material is mandatory and assessment-linked, ensuring that students do not arrive underprepared.*

#### 3.2.3. Self‐Directed Learning (SDL)

SDL is a process where individuals take the initiative, independently or with support, to assess their learning needs, set LOs, identify relevant resources, select and apply suitable learning strategies, and evaluate their progress [14, 97, 98]. In this context, the teacher is the facilitator, not the information source [97]. Implementation of SDL in the early years of health professions education is challenging as it is considered more useful in learners who already have some background knowledge [14, 99]. Managing time effectively while being focused, motivated, and capable of searching learning resources is also imperative to successfully learn with SDL [99]. Instructors at an institution where a revised curriculum incorporated SDL felt that most veterinary students were not prepared for SDL and would prefer traditional lectures, although students performed well in an SDL readiness test [98]. Students in the earlier stages of their education may benefit more from directed self‐learning (DSL), where instructors provide additional support and guidance, and LOs are predefined [14]. Aligning group activities and small group discussions with DSL may also improve student perceptions and academic performance [14]. Completion of a structured logbook consisting of a learning contract and a competency list promotes SDL, although both students and mentors have to commit to completing and reviewing the logbook for maximum utility [100].

SDL is a skill that is particularly relevant to the medical field, as advances are constant and professionals need to become lifelong learners to keep up‐to‐date [101]. More specifically, SDL persistently teaches the utility of reading and learning [99]. The wide range of online resources, including OERs, has added increased opportunities for SDL, although geographical variation and information literacy can complicate this [102]. Although core knowledge may need to be delivered to early learners through other teaching strategies, SDL is an effective method utilized to enhance integration and problem‐solving [18]. When compared with traditional learning methods in a systematic review, SDL was found to be moderately more effective in the improvement of knowledge, skills, and attitudes; this effect was more pronounced when the learners were involved in choosing the LOs [101].

*Veterinary use example: In a Physiology elective, students are given a week where no lectures are held, and they must develop their own LOs focused on a specific physiological mechanism (e.g., renal physiology of a camelid). They define their own LOs, identify peer-reviewed resources to answer their questions, and choose their own method of demonstrating mastery (e.g., creating a recorded podcast or a short review paper). SDL requires students to navigate literature independently, shifting the instructor’s role from a source of truth to a facilitator of information literacy. This technique is recommended for upper-level students or students taking elective courses where they have already mastered foundational concepts. Students must reach a high level of academic maturity to be successful.*

### 3.3. Educational Gaming and Simulation

Over the past 20 years, there has been increasing interest in educational gaming [103, 104]. Initially, games in CD‐ROM format were created and intended to help students review concepts and prepare for class [105], but educational games have evolved considerably with advances in technology and multimedia. Thus far, educational gaming maintains the merits of engaging students, enhancing students’ communication and collaboration, increasing their motivation and critical thinking, and optimizing learning as measured by knowledge acquisition and retention [105–108].

There are several “game”‐related words with different meanings. “Gamification” refers to the use of game elements (e.g., points, leaderboards) in nongame contexts. Extensively applied in marketing contexts, in educational settings, it mainly modifies learners’ behaviors and enhances motivation [109]. Examples of gamification in education include Escape rooms, which are starting to be used in the veterinary field [110]. In contrast, “Serious games” refer to the use of games (with their inherent characteristics, such as rules, mechanics, and elements) for specific educational purposes. “Game‐based learning” (GBL) is a broad‐spectrum term [109], which includes serious games, but has a broader scope, as it includes not only learning by playing, but also by designing and building the games to be played [103]. In order to describe the role of educational gaming in AL, we will separately discuss GBL and simulation. Furthermore, tools in this category are often specific to skills rather than used for generalized instruction.

#### 3.3.1. Game‐Based Learning (GBL)

Availability of GBL resources in the literature varies across health professions fields, with many in medicine focusing on surgery, emergency, or preventative medicine, although topics such as histology are also represented [111]. One video game [10] based on role‐playing adventures helps students master the interpretation of the complete blood count (CBC). Significant differences between students’ pretest and post‐test evaluations and higher final exam scores occurred when students played the game, although differences were more noticeable on the lower range of grades [10]. GBL facilitates deep learning, not only by engaging students, but also by encouraging them to reflect on concepts as they are applying their knowledge in a game scenario [112]. Two interactive games were used as complementary resources for exam preparation for medical and dental students [12]. By answering questions in multiple formats, students could earn virtual “money” to buy virtual prizes at the conclusion while simultaneously getting constant feedback on their answers. The authors observed a significant increase in exam scores, mostly in first‐year students, with positive feedback obtained on surveys [12]. In a similar approach, online free quiz platforms such as Kahoot! (Oslo, Norway) are reported to increase students’ engagement, interest, and collaborative work [113].

Games should follow some principles (Tables 7 and 8), and technology may not be required. For example, crossword puzzle game competitions have been used to review concepts by medical students [114]. Free online resources and apps are available to build them (e.g., https://www.crosswordlabs.com). Crosswords improve knowledge retention and may improve exam scores, and if solved in groups, they improve communication, attitudes, and cooperative learning among students [114]. Even if technology is not a prerequisite for many board and card games, it may facilitate the game process [115], helping to assign points, record achievements, and aggregate results [116], while also meeting some of the expectations of modern students [117] (Table 7).

**TABLE 7:** Elements to consider when designing educational games.

Feature	Recommendation
Feedback	Key feature for learning. Should be immediate and unambiguous
Unpredictable	Different activities with variable complexity/difficulty are more enjoyable
Narrative	Links the game’s interactive elements in a dynamic and engaging experience
Technology	Not mandatory. Effective games (cards, boards) can be simple and low tech.
Individualized	Begin game development with a backward approach (i.e., by defining the learning objectives and establishing students’ needs)
Mock‐up	A prototype should be tested by students without prior experience of that game type
Empower	Involve students in the development process to increase engagement and creativity and best address their preferences
Scaffold	Start with simple questions/activities to increase students’ confidence

**TABLE 8:** Fundamental characteristics of educational games and simulators.

Educational games	Simulators
Fun	Curriculum integration
Motivational	Transfer to practice
Reward system included	Skill acquisition
Feedback (by game or teacher)	Feedback
Competition (disliked by ∼1/3 students)	High‐stakes testing
Collaboration	Team‐training
Technological	Simulation fidelity
Sustainable (i.e., without bugs)	Instructor training and education
Repetition is essential for learning	Deliberate practice
Suitable narratives (adapted to “gamers”)	Mastery learning
Experiential learning	Outcome measurement
Diverse activities	

Recent meta‐analysis studies on GBL have supported their effects on motivation and learning [24, 118, 119] and have highlighted that game quests or missions serve as the most relevant elements for learning [24]. Some authors debate the usefulness of games on learning gains and advise their use as a last resource, when other AL techniques have proved to be ineffective [116]. However, it should be emphasized that GBL should never be viewed as a replacement for a good teacher, but rather as a resource that complements and enriches the learning environment, having its greatest impact on less motivated students [12] (Table 8).

*Veterinary use case: In a Toxicology course, students play a game while taking on the role of a forensic veterinarian. They must identify an unknown toxin based on clinical signs, history, and puzzles that include identifying bits of chocolate in the vomitus. They must subsequently solve a math challenge in order to determine if the dose of theobromine (a stimulant in chocolate) exceeded the toxic threshold. Gaming provides an external narrative structure that maintains high engagement during repetitive or difficult tasks, such as medical mathematics; therefore, it may be particularly helpful for topics with high cognitive loads or when traditional lecturing leads to disengagement.*

#### 3.3.2. Simulation

Simulation has been widely used in aviation, military, and health sciences for decades, with the first anesthesia simulator created in the late 1960s [120] and several models developed ever since [121, 122]. Simulators can include the following: (1) full‐body manikins for practicing several skills; (2) body part models, usually assigned for a single skill; (3) multimedia computer simulations, which may or not include augmented reality features, being used for practical skills or for CBL; (4) virtual reality; (5) scenario‐based simulations, mostly used in the medical field (e.g., role‐playing of simulated patients); and (6) hybrid approaches. The latter includes, for instance, body part models incorporating augmented reality tools [123]. Simulators can range from highly complex and intricate, high‐fidelity models to very simple ones, yet all levels can be useful, since no major differences in skills acquisition have been noted with increasing complexity of models (e.g., in suture skills [124] or collection of cerebrospinal fluid [125]). Some authors also consider ethically sourced cadavers as high‐fidelity simulators [126], but many studies have now shown that model simulators can outperform cadavers for learning purposes [125, 127] and for the transfer of skills to clinical practice, especially for basic techniques [128].

Simulation‐based models have become popular in veterinary education [122, 129, 130] as clinical skills are being introduced earlier in the curriculum [131] and because of the psychological distress of students when practicing painful techniques on live animals [130]. Simulated models offer advantages over real patients, such as their availability for repeated practice without discomfort in a safe and predictable environment for learning, and their variability, allowing replication of different clinical conditions [132]. In simulation, students are the focus of AL, as they are allowed to learn from mistakes, an essential aspect of experiential learning [133].

Studies have highlighted that simulator training reduces the complication rates during “real” clinical and surgical procedures [134, 135], outperforming education lacking simulation or with videos [136], particularly during skill development [124, 137, 138]. Furthermore, simulation has been shown to be more effective than CBL for teaching certain concepts [61]. The performance of FNA skills is similar when the procedure is taught in live animals or in manikins [139, 140].

**FIGURE 3:**
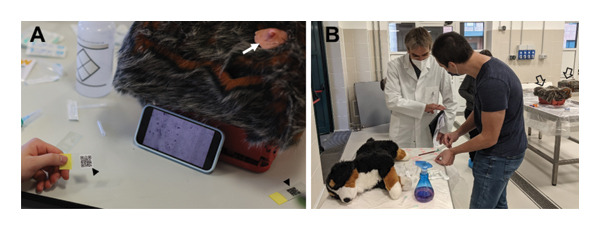
Simulation procedures in clinical pathology. (A) After collecting samples from simulation models representing various lesions, students review corresponding microscopy videos on their mobile devices. These videos are accessed via QR codes embedded on the slides (arrowheads). (B) Incorporating an objective structured clinical examination (OSCE) station into simulation‐based training is essential. After practicing on models (open arrows), the student collects specimens from a realistic simulated case and receives individualized feedback from the instructor. This step is critical for reinforcing skills and promoting effective learning.

Augmented and virtual reality can simulate various scenarios. Augmented reality refers to layering virtual objects over the real environment, and it only requires a smartphone or tablet. Virtual reality establishes a fully virtual environment, requiring a computer with interactive 3D visualization and a head mount display or 3D glasses, often coupled with gloves with position trackers. These tools are widely used in simulation settings, since they can enrich or fully recreate scenarios that are hard to deliver in the real world [141]. Augmented reality tools, accessed through quick response (QR) codes, have been used for teaching cytology sample collection (Figure 3A) [123], creating an immersive simulation with virtual cases and microscope slides, coupled to the real collection of material from models [123, 140]. The variety of scenarios and roleplay activities that can be created through virtual reality is immense, and virtual simulations can be conducted with fewer resources compared to physical ones [141].

McGaghie et al. [142] outline traits simulators should have, though meeting all criteria is challenging (Table 8) [130, 143]. Feedback, especially from the simulator or postsimulation, is crucial for deeper learning [144], enabling self‐assessment and preventing bad habits [145, 146]. Peer feedback also reinforces learning, and debriefing is crucial for knowledge consolidation [142]. Outcome measurement, often through objective structured clinical examination (OSCE) tools [142], is essential. Some OSCE testing needs to be performed in specific settings such as a laboratory [145] or may require additional personnel (Figure 3B); the latter requirement may be circumvented by technology (e.g., students record the procedure and upload the video for evaluation) or by creative solutions (e.g., students evaluate their classmates).

*Veterinary use case: In a Critical Care course, students use a canine mannequin to manage a sudden cardiac arrest. The mannequin is equipped with sensors that provide real-time feedback on compression depth and rate, intubation success, and heart rhythms that the instructor can change remotely to reflect the patient’s declining or improving status. Students practice performing chest compressions, selecting the appropriate drugs and drug dosages, and using clear communication. Simulation can be used to teach skills where errors in a live setting could lead to patient discomfort or negative outcomes, including death. Costs can vary depending on the type of simulator used, and the less expensive versions often require some time investment to build. Depending on the skill represented, simulators may be useful for scenarios ranging from entry-level clinical skills to advanced, capstone training labs.*

## 4. Important Considerations

Veterinary curricular changes in response to the CBVE framework have highlighted the paramount role of students, and the emphasis has shifted from “content” to “performance.” While increasing class sizes, budget constraints, faculty turnover, and changes in postpandemic student populations may also be contributing to these curricular changes, veterinary teaching faculty are being asked to replace passive lectures and teacher‐centered scenarios with AL. While empirical studies are relatively easy to find in the literature, our literature review confirmed that few focus specifically on veterinary medicine and most rely on surveys to measure outcomes, without appropriate control groups. This disproportionate focus contrasts with recent and ongoing curricular changes in veterinary education. The current lack of robust quantitative evidence on the effectiveness and impact of AL in veterinary medicine highlights the need for further research in this area.

While more investigation is needed, this review has summarized many AL options that vary in time investment and accessibility to technology, among other features. In a study focused on first‐year medical schools in the US, the most used methods of AL were as follows: discussion/debate, CBL, ARS, and formative quizzes [21], all of which are described herein. Given the variability in individual instructor preference, institutional priorities, and access to specific techniques, we attempted to provide a comprehensive resource offering useful and adaptable options for diverse educational settings. As the purpose of this article was not to challenge the validity of the included descriptive studies, perceived advantages and limitations were often reported to help instructors make informed decisions when considering new teaching methods. As AL tools like GBL [104] can engage and motivate students by fostering a fun learning environment and supporting the development of learning communities [116], their implementation, at least as a supplemental teaching tool, appears reasonable.

Still, as most educators recognize, no single pedagogical strategy should be viewed as a panacea for effective teaching across all student populations. As with any educational intervention, it is best to begin with clearly defined LOs and then select one or a combination of AL methods accordingly. When implementing AL strategies, both student and instructor time should be carefully considered. It is well recognized that medical knowledge and textbooks continue to expand, whereas curricular hours, especially in basic sciences, have been reduced due to curricular slimming [147]. Consequently, strategies such as the FC may seem enticing as an opportunity to teach the same amount of content with fewer classroom hours. However, new FC adopters must consider the typically intensive schedules of veterinary students, as well as the growing emphasis on student and faculty well‐being, before developing extensive preparatory materials that require significant out‐of‐class time.

Blended learning with a balance of AL and passive learning is required. Such an approach is anticipated to maximize the benefits and minimize the limitations of both approaches. To some degree, this balance may be curated by the curricular development process as didactic (lecture) time is compressed and purposefully scheduled near integrative sessions and laboratory periods. Instructors, particularly those charged with leading the didactic time, are encouraged to consider integrating or implementing AL techniques to promote student engagement and reflection.

A primary limitation of this review concerns the scope of the initial search strategy. By utilizing the overarching terms such as “active learning,” “active teaching,” “student centered,” and “student focused” as primary search criteria, rather than conducting secondary searches for each of the subcategories, the study may have omitted specialized papers that do not explicitly use the “AL” nomenclature. Consequently, while this review provides a robust synthesis of the broad field, we may have missed specific niche applications of individual methodologies, particularly those in the veterinary medical field. However, this search strategy was purposefully designed to use broad terms to establish a comprehensive baseline and a global status update of the field.

Additionally, the authors would like to note the lack of content on generative AI and adaptive learning platforms. The formal literature search for this review was performed in October 2023, and the majority of peer‐reviewed research on generative AI and adaptive learning platforms has been published or indexed after this time. As a result, specific activities using these tools fell outside the scope of data collection. These emerging technologies are likely to be a priority of future investigations.

## 5. Conclusion

The list of AL techniques is lengthy, and the literature in this field is expanding. Most reports are outside the realm of veterinary medicine, and typically, only one method or intervention is described in each article. This summary highlights several different techniques, describes how they have been or could be used, and lists some of their perceived advantages or limitations. As a result, it serves as a starting point for veterinary educators to expand their knowledge and use of AL in the changing curricular environment. It is best practice to delineate the status of AL in veterinary medicine, so that more quantitative evidence of its effectiveness and impact in this field can be obtained in the future.

## Author Contributions

Project conceptualization came from Bridget C. Garner. All authors developed the research focus and methods, contributed to the search, and wrote the original manuscript. Bridget C. Garner edited the final version for submission.

## Funding

The research project on which this paper is based was funded by an American Association of Veterinary Medical Colleges Council on International Veterinary Medical Education (AAVMC CIVME) Educational Exchange grant.

## Disclosure

All authors have approved the manuscript for submission to publication.

## Conflicts of Interest

The authors declare no conflicts of interest.

## Supporting Information

Additional supporting information can be found online in the Supporting Information section.

## Supporting information


**Supporting Information 1** Inclusion and exclusion criteria for references.

**Supporting Information 2** Glossary.

## Data Availability

The articles used in this review have been appropriately credited and cited. The extracted data from chosen articles supporting the findings of this review are presented within the manuscript and are available from the corresponding author upon reasonable request.
