# Supplementary material for: Active Learning Strategies in Veterinary Medicine: A Literature Review
Source: Vet Med Int. 2026 Aug 4;2026:7397383. doi: 10.1155/vmi/7397383 (PMC13438178; doi:10.1155/vmi/7397383)
Supplement: Supplementary file 1 [file VMI-2026-7397383-s001.pdf]

Glossary

Term	Definition
Active learning	Educational activity where students engage and interact with the learning process. It incorporates intentional engagement, purposeful observation and critical reflection, and it encourages faculty and students to be cognitively active in the classroom
Audience response systems (ARS, or personal response systems or electronic voting systems)	Systems which allow large groups to answer questions, often anonymously, in real time during lectures
Augmented reality	A technology that superimposes digital information (such as images, models, or instructions) onto the real-world environment, enhancing interactive learning.
Blended learning	Combines traditional face-to-face instruction with online content
Case modelling	An instructional method in which the instructor explicitly verbalizes their clinical reasoning and decision-making processes during the management of a case, helping students understand the cognitive steps involved.
Case-based learning (CBL)	A structured educational approach that presents students with clinical scenarios to apply their prior knowledge, typically of disease pathophysiology, helping to develop problem-solving skills without requiring direct patient contact.
Directed self-learning (DSL)	A structured form of self-directed learning in which learners engage with material independently but within a framework provided by instructors, including predefined learning objectives, guided resources, and ongoing support.
Flipped classroom (FC)	An instructional approach that delivers theoretical content before class, using in-person sessions for active learning strategies such as discussion, group work, problem-solving, and reflection to deepen understanding.
Game-based learning (GBL)	An instructional approach that uses game elements and principles to create engaging, interactive learning experiences, with the aim of enhancing understanding, motivation, and knowledge application.
Gamification	The use of game elements (e.g., points, leader boards) in non-game contexts
Jigsaw scheme	A collaborative learning strategy where each student is assigned a unique topic to master and then teach to their group, promoting active engagement and peer instruction.
Muddiest point	An activity in which students, often anonymously, indicate which topic or concept they find most confusing, providing instructors with targeted feedback for clarification.
Passive learning	An instructional approach in which students receive information from an instructor without active engagement, often through

	methods such as lectures or reading, with limited opportunities for interaction, discussion, or immediate application
Peer-to-peer learning / peer-assisted learning	A learning process in which students learn with and from their peers, often through discussion, collaboration, and reciprocal teaching.
Problem-based learning (PBL)	A student-centered approach in which learning begins with a complex problem, prompting students to identify what they need to learn and actively seek out relevant information to solve it.
Self-directed learning (SDL)	A process in which individuals take initiative, with or without guidance, to assess their learning needs, set goals, identify resources, choose strategies, and evaluate their progress.
Serious games	Use of games (with their inherent characteristics, such as rules, mechanics and elements) for specific educational purposes.
Simulation	An instructional method that replicates real-world clinical or procedural scenarios through models, technology, or role-play, enabling learners to practice skills, make decisions, and learn from mistakes in a safe, controlled, and repeatable environment, supporting skill development, critical thinking, and team-based decision-making.
Surface learners	Learners who focus on memorization rather than deep understanding, often aiming to meet minimum requirements rather than engage meaningfully with the material.
Teacher-centered	An instructional model in which the teacher is the primary source of knowledge and authority, directing the learning process through lectures, demonstrations, and structured activities, with students typically playing a more passive, receptive role
Team-based learning (TBL)	A small-group teaching method in which stable teams work together over time through a recurring sequence of activities that promote accountability and collaborative problem-solving.
Think-pair-share (TPS)	A collaborative in-class activity in which students first reflect individually on a question, then discuss their ideas with a partner, and finally share their conclusions with the whole class
Two-envelope method	A blended learning activity where students complete a pre-class reading, take an individual quiz in class, then reattempt the same quiz with added clinical context in groups, followed by a faculty-led discussion.
