# Supplementary material for: Active Learning Strategies in Veterinary Medicine: A Literature Review
Source: Vet Med Int. 2026 Aug 4;2026:7397383. doi: 10.1155/vmi/7397383 (PMC13438178; doi:10.1155/vmi/7397383)
Supplement: Supplementary file 2 [file VMI-2026-7397383-s002.docx]

Inclusion and Exclusion Criteria

Inclusion:

- Interventional or descriptive studies using active learning techniques
- Reviews on active learning
- Instructor experiences with active learning, including challenges and benefits
- Health professions curriculum (veterinary, dental, medical, pharmacy and nursing) and other adult degree programs in science-related fields (e.g. undergraduate biochemistry, biology,) using active learning

Exclusion:

- Published prior to 2003
- Not published in English
- Full text not available online
- Not peer-reviewed (limited in ERIC database only)
- Use of “active learning” meant to indicate computerized or machine learning techniques
- Primary focus on comparing remote to in-person learning
- Child learning
- Summaries of health professions curricular models
- Insufficient detail of active learning method to either understand or recreate
- Primary focus on student metacognitive processes
- Technique not adaptable to veterinary medicine
